# Olfactory neurofeedback: current state and possibilities for further development

**DOI:** 10.3389/fnhum.2024.1419552

**Published:** 2024-11-29

**Authors:** Ivan Ninenko, Alexandra Medvedeva, Victoria L. Efimova, Daria F. Kleeva, Marina Morozova, Mikhail A. Lebedev

**Affiliations:** ^1^Institute for Cognitive Neuroscience, HSE University, Moscow, Russia; ^2^Pedagogy Department, International University of Central Asia, Tokmok, Kyrgyzstan; ^3^V. Zelman Center for Neurobiology and Brain Rehabilitation, Skolkovo Institute of Science and Technology, Moscow, Russia; ^4^Developmental Psychology and Family Pedagogic Department, The Herzen State Pedagogical University, Saint Petersburg, Russia; ^5^MSU Institute for Artificial Intelligence, Lomonosov Moscow State University, Moscow, Russia; ^6^Sechenov Institute of Evolutionary Physiology and Biochemistry of the Russian Academy of Sciences, Saint Petersburg, Russia; ^7^Faculty of Mechanics and Mathematics, Lomonosov Moscow State University, Moscow, Russia

**Keywords:** olfaction, EEG, BCI, NFB, olfactory processing

## Abstract

This perspective considers the novel concept of olfactory neurofeedback (O-NFB) within the framework of brain-computer interfaces (BCIs), where olfactory stimuli are integrated in various BCI control loops. In particular, electroencephalography (EEG)-based O-NFB systems are capable of incorporating different components of complex olfactory processing – from simple discrimination tasks to using olfactory stimuli for rehabilitation of neurological disorders. In our own work, EEG theta and alpha rhythms were probed as control variables for O-NFB. Additionaly, we developed an olfactory-based instructed-delay task. We suggest that the unique functions of olfaction offer numerous medical and consumer applications where O-NFB is combined with sensory inputs of other modalities within a BCI framework to engage brain plasticity. We discuss the ways O-NFB could be implemented, including the integration of different types of olfactory displays in the experiment set-up and EEG features to be utilized. We emphasize the importance of synchronizing O-NFB with respiratory rhythms, which are known to influence EEG patterns and cognitive processing. Overall, we expect that O-NFB systems will contribute to both practical applications in the clinical world and the basic neuroscience of olfaction.

## 1 Introduction

BCIs are a multidisciplinary field that holds the promise to revolutionize medicine, technology, and science through the development of systems that bidirectionally connect the nervous system to external devices (Lebedev and Nicolelis, 2017). Neurofeedback (NFB) systems are a subtype of BCIs that convert neural signals into stimuli delivered to human senses to enable self-regulation (Marzbani et al., 2016; Hammond, 2011; Sitaram et al., 2017). Historically, NFB systems have relied mostly on visual and auditory modalities. Here we discuss the idea of using olfaction for NFB.

We suggest that the effects of O-NFB could be unique compared to other NFB types because of the distinct features of odor processing in the brain. The olfactory inputs to the brain originate in the olfactory epithelium of the nose where olfactory neurons respond to odors and send their outputs to the olfactory bulbs (Morrison and Costanzo, 1990). The olfactory bulbs, unlike the other sensory systems, do not connect to the thalamus but project directly to the olfactory cortex (Shepherd et al., 2004). The olfactory bulbs also connect to the hypothalamus, amygdala, entorhinal cortex, and ventral striatum (Wilson and Mainen, 2006) which could be considered as limbic regions (Gottfried, 2006) where emotions are processed (Sullivan et al., 2015; Soudry et al., 2011) including the emotions in the context of social interactions (Sullivan et al., 2015; Malaspina et al., 2006). The projection areas of the olfactory bulb connect to the mediodorsal thalamus, orbitofrontal cortex, and hippocampus (Courtiol and Wilson, 2015). This distributed connectivity of the olfactory signals enables an important role of olfaction in cognition (Richardson and Zucco, 1989; Stevenson, 2013). Thus, olfactory memories have a profound emotional impact, surpassing those elicited by other sensory stimuli (Delaunay-El Allam et al., 2010; Carskadon and Herz, 2004). Disorders of olfaction contribute to such neuropsychiatric conditions as anxiety (Burón and Bulbena, 2013), depression (Kohli et al., 2016), schizophrenia (Marin et al., 2023) and bipolar disorder (Kazour et al., 2017). Additionally, olfactory dysfunctions occur in neurodegenerative diseases such as Parkinson's disease (Doty, 2012) and Alzheimer's disease (Murphy, 2019). Olfactory dysfunctions are also present in autism spectrum disorders (ASD) (Marin et al., 2023; Tonacci et al., 2017). Children with ASD have been reported to have reduced odor identification and discrimination abilities, where the severity of ASD symptoms is correlated with the ability to identify odors (Sweigert et al., 2020; Rozenkrantz et al., 2015), but some authors have disputed these findings (Dudova and Hrdlicka, 2013; Larsson et al., 2017). The COVID-19 endemic has introduced new challenges to the treatment of olfactory dysfunctions (Hannum et al., 2020).

Notwithstanding the previous research on the neural mechanism of olfaction and neural disorders affecting olfaction, the human sense of smell is still much less studied compared to the other sensory modalities, particularly vision (Hutmacher, 2019). There is still no consensus on the classification scheme that should be used for odors. Yet, humans possess an excellent olfactory ability, and the notion that their sense of smell is weak compared to other animals is clearly a isconception (McGann, 2017) even though, many people find it challenging to describe a smell in words (Yeshurun and Sobel, 2010; Distel and Hudson, 2001) or they rank the sense of smell as the least important (Enoch et al., 2019). The olfactory modality is currently underappreciated in neural technologies, even though supporting technologies have been developing such as the electronic nose (Wilson and Baietto, 2011).

## 2 The challenges of olfactory neurofeedback

In this perspective, we build on our results to propose that the O-NFB approach should be expanded in the future to give rise to the next generation of medical and consumer applications that integrate neural recordings with olfactory displays. Olfactory neurofeedback has several potential applications and multiple challenges. Olfactory dysfunctions (Croy et al., 2014) are the primary area of application for O-NFB. Olfactory training, also known as smell training or scent training, has already proven effective in restoring or improving the sense of smell in individuals with olfactory impairments (Hummel et al., 2009; Sorokowska et al., 2017). Although using olfaction as NFB appears as a reasonable idea, implementations are not as straightforward as with visual and auditory NFB. The complexity of olfactory stimulation (Lorig, 2000) and the methodological challenges of obtaining a reliable neural marker of olfaction (Masaoka et al., 2014) have kept this sensory system outside of the mainstream research on NFB.

Given the abundance of EEG-based approchaes to both BCI (Abiri et al., 2019) and NFB (Omejc et al., 2019), EEG recordings could be the easiest way to implement an O-NFB. EEG research has been ongoing of olfactory processing with such approaches as evoked potentials (Lorig, 2000; Livermore et al., 1992; Arpaia et al., 2022) and EEG rhythms (Schriever et al., 2017; Aydemir, 2017; Cherninskii et al., 2009). EEG modulations have been linked to such characteristics as odor pleasantness (Abbasi et al., 2019; Kroupi et al., 2014; Kim and Watanuki, 2003) and relationship to food (Martin, 1998). Notably, olfactory stimulation induces an increase in EEG power in the theta-rhythm range in frontal-temporal areas (Morozova et al., 2023; Schriever et al., 2017; Hucke et al., 2021). Theta and alpha rythmes modulations have been also linked to emotional responses during olfactory perception (Martin, 1998; Brauchli et al., 1995; Cherninskii et al., 2009), and alpha-rhythm modulations have been reported in a study that combined functional magnetic resonance imaging with magnetoencephalography (Tarfa et al., 2023). Attention to olfactory stimuli modulates EEG in the inferior frontal cortex, insula, and inferior temporal gyrus (Singh et al., 2019). Additionally, when mice explore odors and objects, correlation between EEG in the delta and theta range and their respiration decreases (Dasgupta et al., 2024). Machine learning algorithms have been applied to discern EEG patterns elicited by different odors (Ninenko et al., 2021; Aydemir, 2017; Saha et al., 2014).

In parallel to the studies of EEG responses to odors and the decoding of odor identity from EEG, there has been work toward solving the significant technical hurdles in eliciting and delivering olfactory stimuli that would be needed for an effective O-NFB system. The appropriate odor-delivery system should be capable of both precise delivery of scents and their removal. Such properties of olfactory responses as persistence, multiplicity, and masking complicate the transition between different scents and potentially lead to interference (Yanagida, 2008). Odors are complex mixtures of volatile molecules that can vary widely in their composition and concentration. This variability makes it challenging to precisely control odor stimuli across experiments. Odor perception is variable across individuals (Hudson and Distel, 2002; Trimmer et al., 2019; Wright and Mitchell, 2005) which makes it challenging to establish a standardized set of odor stimuli suitable for all participants. Addressing these challenges is essential, especially when rapid scent changes are required without causing an overlap or confusion of different olfactory inputs. Several odor-delivery methods have been developed (Yanagida, 2012; Wen et al., 2018; Garcia-Ruiz et al., 2021). One method involves scented waxes or oils that release odors upon air exposure. The other method uses compressed scent in bottles, sprayed into the air with an electronically controlled device. The third method consists of encasing scents in cartridges and dispersing them via inkjet technology for precise scent delivery (Sugimoto et al., 2010). In the fourth method, a scented mist is produced using ultrasonic transducers. This mist is added to the stream of air.

Regarding the methods for odor delivery to the olfactory epithelium, odor containing air could be delivered to participants either be spraying them in front of the nose or through the use of airtight masks, which are commonly employed for controlled olfactory stimulation. These masks create a sealed environment that isolates specific odorants and mitigates contamination be the odors from external sources. Odor accumulation within a mask is a limitation of this method. As participants inhale and exhale, odors accumulate and residual odors could interact with the olfactory stimuli, causing variability in the perception of odor intensity and quality. Given this limitation, spraying odors in a well-ventilated room could be a better solution than wearing a mask. In this room, precautions should be taken to minimize the effects of olfactory cross-contamination when multiple odors are utilized. Surfaces that could come into contact with odorants should be cleaned with an alcohol solution between participants. It is important to note that odor delivery devices could retain residual scents even after a single experimental session; therefore, regular cleaning of the surfaces in contact with odorants is essential to ensure the fidelity of the experimental conditions.

## 3 Specific experiments with O-NFB and their implications

Notwithstanding the improvements in our understanding of how olfactory processing is manifested in EEG, technological developments in the olfactory displays have already laid a foundation for olfactory-based BCIs and O-NFB systems. Yet, very little progress has been made in such neural technologies. Besides our own writings on these topics (Morozova et al., 2023; Ninenko et al., 2021; Medvedeva et al., 2024), there is just one human study of an olfactory-based biofeedback (Miyaura et al., 2011), where participants' level of concentration was controlled. A custom-built olfactory display was mounted under the nose to provide feedback of the concentration level measured based on the electrocardiogram analysis. The odors presented this way improved the performance, but constant odor delivery was effective, as well. In animal research, an olfactory-based BCI has been mentioned (Dong et al., 2014) but no real-time system has been developed.

**Figure 1:**
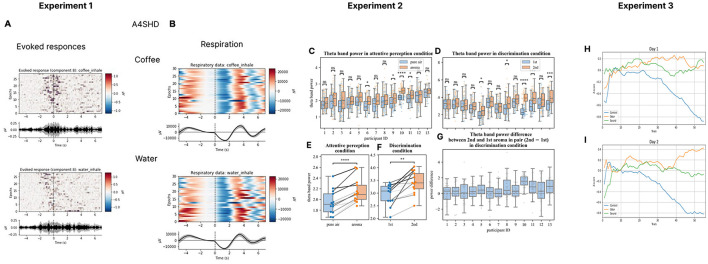
EEG recordings during olfactory stimulation and O-NFB. Evoked responses **(A)** and respiratory data **(B)** for coffee and water smells for one participant during Experiment 1 where a version of then olfactory instructed-delay task was implemented was implemented (Ninenko et al., 2021). The analyses are also depicted of the changes in theta power **(C, D, G)** and the across-averaged for each participant average theta power in perception **(E)** and discrimination **(F)** tasks from Experiment 2 where participants perceived and memorized odors or discriminated pairs of odors (Ninenko et al., 2021). Additionally, floating-mean progression of z-cored alpha power the change is shown for day 1 **(H)** and day 2 **(I)** of O-NFB training. The floating mean is shown for the Z-score of the alpha-range PSD envelope. Day 1 **(H)** and day 2 **(I)** data are shown from experiment 3 where an O-NFB was implemented based on the EEG alpha rhythm.

Based on our work, we have recently proposed a research program aimed at the development of olfactory-based BCIs (Morozova et al., 2023). Toward this goal, we have conducted several studies where EEG was recorded in settings suitable for an O-NFB (Experiment 1 in Figures 1A, B, Table 1). Ninenko et al. (2021) developed an instructed-delay paradigm to investigate EEG modulations during olfactory discrimination. An automated odor delivery system was utilized to ensure a precise and controlled presentation of olfactory stimuli and respiration data. The olfactory delivery system was mounted on the ceiling of a custom-designed room. A constant airstream descended from above, into which odors were seamlessly integrated. The instructed-delay task required associating specific odors with visual targets and reporting perceived odors by pointing to those targets with a joystick. The participants held their breath before the odorant delivery, which ensured a consistent breath cycle during the task execution. The odor-perception delay was 10 s, which corresponded to 2-3 breath cycles. The participants had a median success rate of 93.8% for odor discrimination and the participants' ability to differentiate scents did not change over a one-hour session. With this approach, we obtained insights into the spatial and spectral properties of cortical activity associated with olfactory processing. In particular, a ~12 Hz rhythmic activity was observed over the EEG recording site C4, which was sensitive to olfactory processing. This ~12 Hz rhythmic activity falls into the high-alpha or low-beta frequency range (Egner and Gruzelier, 2004). The latter has been linked to attentive processing and sensorimotor functions (Gola et al., 2013; Kilavik et al., 2013). Given the involvement of respiratory modulation in olfactory tasks, it is plausible that this activity was related to the sensorimotor processes essential for odor discrimination. Additionally, the visually evoked response P200 was modulated depending on the perceived odor. We suggest these EEG modulations, combined with data from studies on olfactory event-related potentials (Invitto and Mazzatenta, 2019; Rombaux et al., 2007), should be further explored for possible use in O-NFB systems.

**Table 1:** EEG recordings during olfactory stimulation and O-NFB.

**Description of the experiments**						
**Experiment**	**Odor delivery**	**Task**	**Subjects, trials**	**Inhalation cycle**	**Odors**	**Results**
Experiment 1. An experimental paradigm for studying EEG correlates of olfactory discrimination (Ninenko et al., 2021)	Piezoelectric transducers were used to release liquid odorants into the air stream. Airflow was driven from the ceiling to the floor by a set of fans.	Discrimination of odors based on abstract symbols (not odor-related)	N = 17 subjects. Training: 40 trials, 10 × odor, discr.: 80 trials, 20 × odor. About an hour, depending on the participant	Regulated by the participant. Each trial is initiated by a button. Holding breath for 2 s.	Vanilla, coffee, citrus, water (control stimuli)	1. Olfactory-related responses peaking around 12 Hz were detected over the C4 electrode. 2. Subjects successfully discriminated odors through the experiment
Experiment 2. An olfactory-based Brain-Computer Interface: electroencephalography changes during odor perception and discrimination (Morozova et al., 2023)	Aroma Shooter diffuser. The diffuser was equipped with six aroma cartridges	Discrimination of odor pairs	13 subjects. Training: 100 trials (17 min session). Discr. 50 trials. (20 min session)	Inhalation and exhalation according to auditory commands	Caramel, grass, orange, pine, smoke, and mint	Changes in frontal theta power during odor inhalation and discrimination.
Experiment 3. The development and testing of olfactory-based neurofeedback for the EEG alpha rhythm (Medvedeva et al., 2024)	Robotic display carried out the supply of odor by raising from a sealed tube and holding aromatic markers in front of a fan blowing away the smell in the subject's nose.	To increase alpha power using neurofeedback.	*N* = 15. Three groups: audio, olfactory and mock feedback. Two days with 20 min sessions. Feedback was provided for 10 s, every 10 s.	Inhalation after tone played each 10 s	Standard marker Sniffin' Sticks with banana flavor	Increase in the alpha range PSD in olfactory and auditory feedback, followed by a decline and a stable level maintained until the end of the session. Gradual, constant decline in the control group.

Morozova et al. (2023) (Experiment 2 in Figures 1C–G, Table 1)utilized a somewhat different experimental arrangement to explore the EEG manifestation of odor perception and discrimination. The Aroma Shooter diffuser (developed by Aromajoin Corporation, Japan) was used for odor delivery. This device generated 0.5 s long olfactory stimuli which the participants perceived and memorized or discriminated. In the discrimination task, a sequence of two odors was presented, and the participants reported by pressing a keyboard key whether they were the same or different. With this methodology, we found modulations in the frontal EEG theta (4-7 Hz) evoked by the olfactory stimuli. Additionally, an increase in the parietal alpha power (8-12 Hz) was observed during odor memorization.

Medvedeva et al. (2024) (Experiment 3 in Figures 1H, I, Table 1) proceeded with the development of an olfactory-based NFB system for controlling the EEG alpha rhythm (8-12Hz). In this study, odors were delivered using a robotic device that positioned Sniffin' Sticks markers in front of the participant's nose. The participants were divided into three groups by the type of feedback they received: O-NFB, auditory NFB, and mock-olfactory NFB. During each 20-minute session, subjects kept their eyes closed and received NFB of their alpha rhythm every 10 s. The O-NFB and auditory NFB participants received the corresponding reinforcement if their alpha rhythm power exceeded a predefined threshold. Two daily sessions were conducted per participant. The study showed comparable increases in alpha power in the O-NFB and auditory NFB groups but not in the mock NFB group. Overall, this study is a proof of concept demonstration of the feasibility of an EEG-based olfactory NFB system.

## 4 Discussion

While our work on O-NFB is currently at a proof-of-concept stage, we foresee a bright future for O-NFB systems and suggest that they have significant potential for multiple medical and consumer applications, including neural technologies that improve olfactory training (Ojha and Dixit, 2022; Hummel et al., 2009; Sorokowska et al., 2017) rehabilitate emotions (Taalman et al., 2017), enrich memory (Wilson and Stevenson, 2003), and augment cognition (Chen et al., 2022).

Based on our experience in the experiments involving olfactory stimuli, we offer several recommendations for the researchers entering this field. Firstly, when analyzing evoked brain activity in response to olfactory stimuli, one should consider not only the timing of stimulus presentation but also the respiratory cycle. This consideration is particularly important for the settings where the stimulus onset does not align with the onset of inhalation. To mitigate this, the respiratory cycle should be monitored using appropriate sensors. Additionally, external cues (e.g. visual or auditory stimuli) could be added to guide inhalation and exhalation. Secondly, we recommend using a control where respiratory cycles occur without odorant inhalation. Since respiratory cycles itself change brain activity (Heck et al., 2017), data from the odorless respiratory cycles would help to elucidate the specific contribution of olfactory stimuli to neural modulations when stimuli are presented. Thirdly, in analyzing both evoked and induced brain activity, eye position and state (open or closed) should be monitored and ocular artifacts should be removed from the electrophysiological data. Participants often close their eyes during aroma inhalation and exhibit eye movements when engaged in cognitive tasks. These eye movements could be confused with the evoked potential. Additionally, eye closure could elevate alpha power, potentially leading to erroneous interpretations. Depending on the specific experimental protocol, it could be advisable to instruct participants to keep their eyes closed throughout the session to reduce ocular artifacts. Fourthly, researchers should systematically collect additional behavioral data regarding participants' subjective experiences caused by the presented aromas. It is important to gather information on the perceived intensity of the olfactory stimuli and their specific features. Olfactory performance could be evaluated prior to the experiment using tests, such as the Sniffin' Sticks test (Hummel et al., 1997).

Given that O-NFB systems currently do not exist, a reasonable course of their emergence and development would be by mimicking and supplementing the capacity of the existing visual and auditory NFB systems, which are abundant because of the ease of their implementation. Since O-NFB is technically more challenging, the realization of its potential hinges upon addressing several critical factors inherent to olfactory feedback delivery. Foremost among these is the precise presentation of olfactory stimuli independently of the concurrent sensory modalities. To achieve this, odor delivery protocols should be used that maintain consistent airflow, facilitate the swift removal of odors post-delivery, and prevent residual scents from affecting subsequent stimuli. The development of olfactory displays has been impressive in recent years (Garcia-Ruiz et al., 2021) including the ones integrated with virtual reality (Liu et al., 2023; Micaroni et al., 2019), which ensures that the needs of the specific O-NFB systems can be met.

One advantage of the current visual and auditory NFB systems is their capacity for feedback delivery with a short delay. For instance, NFB of the parietal alpha rhythm works better when delivered promptly (Belinskaia et al., 2020) and the shorter the BCI delay the greater is the sense of agency (Evans et al., 2015). Electrophysiological responses to odors can occur as early as 200 ms after the stimulus, as demonstrated in the studies of olfactory event-related potentials (Invitto and Mazzatenta, 2019; Rombaux et al., 2007). However, perceptual decisions are typically slower, taking approximately 1 s (Olofsson, 2014). Additionally, olfactory perception occurs only during inhalation, making it discontinuous. Therefore, O-NFB systems should account for the respiratory cycle and synchronize olfactory displays to the readings of respiration. It should be also considered that such synchronization could lead participants to modify their breathing patterns to optimize O-NFB outcomes – the effect that could be desirable or undesirable depending on the application.

The other consideration for the O-NFB systems is that their performance could improve if recordings are conducted from the primary olfactory structures such as the olfactory bulb and olfactory cortex. Even though this is a difficult task for EEG recordings, electrode placement on the forehead allows picking up the signal from the olfactory bulb (Iravani et al., 2020, 2021; Cakmak et al., 2020). O-NFB could be made more efficient when integrated with invasive recording methods such as stereoelectroencephalography (sEEG), which enables direct recordings from the olfactory cortex (Yang et al., 2022).

The potential applications are numerous where O-NFB could target specific olfactory functions or dysfunctions. To mention a few, O-NFB could be instrumental in olfactory rehabilitation following conditions like COVID-19 (Hannum et al., 2020) stress management recovery after cessation of smoking (Ajmani et al., 2017), and brain health for the elderly (Chen et al., 2022). O-NFB is potentially applicable to the treatment of autism spectrum disorders (ASD), where the peculiarities of the sense of smell could contribute to the eating disorders in ASD. O-NFB could become a tool for olfactory training in professions that rely heavily on a well-developed sense of smell, such as sommelier (Filiz et al., 2022). Additionally, exploiting olfaction's unique characteristics, such as its influence on perception of the other sensory modalities, could augment the current NFB approaches like visual and auditory NFB. Unlike visual or auditory NFB, which typically require awareness and attention, O-NFB could work without these requirements but still influence cognitive and emotional states. Such covert delivery of O-NFB would enable a seamless integration of olfaction with the other sensory modalities to exert slow modulations of the faster NFB mechanisms.

O-NFB systems present a number of ethical challenges. In medical applications, privacy and informed consent are paramount, as brain activity data could be highly sensitive. It is crucial to ensure that participants understand how their neural data will be used. In consumer settings, the potential misuse of NFB systems, whether for manipulation cognitive states or tracking data without consent, should be controlled for. Overall, the responsible development and regulation of O-NFB systems will be essential for ensuring ethical application of this technology.

Exploring the feasibility of administering olfactory stimuli during sleep stages presents another avenue for future research (Badia et al., 1990; Barnes and Wilson, 2014). Olfactory stimuli could be delivered during sleep without disturbing the person (and stimuli of the other modalities would be more disturbing) and this delivery could be aligned with particular EEG phases and the stages of sleep. An O-NFB could modulate sleep patterns this way, which could work as a treatment for sleep disorders.

Personalization is an important consideration for optimizing O-NFB protocols. Accounting for features like individual EEG characteristics, electrode positions, individual responses to odors, and task difficulty could improve NFB effectiveness. For example, allowing participants to choose a specific set of odors could improve the intervention's efficacy. While olfaction is inherently subjective and influenced by prior experiences, our previous work (Ninenko et al., 2021) has demonstrated low variability in discrimination performance regardless of prior experience. In these experiments, individual variability was observed in the ability to correctly name odors. However, the sample was relatively small and we could not relate this variability to the subjects' cultural background. Moving forward, researchers should choose between further standardization of the olfactory settings vs. individualization depending on the experimental objectives.

O-NFB, which is the system where a smell is generated based on the analysis of neural activity, is not the only possible olfactory BCI system. The other class of olfactory-based BCIs could utilize odor stimuli as the initial inputs that drive the BCI operations. For instance, olfactory training could consist of odor delivery followed by the selection of a matching visual image using a P300 BCI (Morozova et al., 2023). Such a BCI could be useful for the rehabilitation of olfactory dysfunctions and for the rehabilitation of dementia, as well.

Finally, the sense of smell could be evoked by the electrical stimulation of olfactory structures (Holbrook et al., 2019; Karunanayaka et al., 2023) and the orbitofrontal cortex (Bérard et al., 2021), and such stimulation could be considered a bidirectional BCI or a form of O-NFB, with a range of medical applications. These findings highlight the potential for using neuromodulation techniques to artificially induce olfactory sensations, which could be applied to BCIs and O-NFBs for sensory restoration or enhancement. In particular, electrical stimulation of the olfactory bulb has been shown to evoke smell perception in individuals with olfactory dysfunction, building expectation of rehabilitation in conditions such as anosmia (Holbrook et al., 2019). Furthermore, stimulation of cortical areas like the orbitofrontal cortex can elicit pleasant olfactory experiences, suggesting that such interventions could not only improve olfactory function but also modulate emotions and reward-related processing (Bérard et al., 2021).

However, there are notable limitations to this approach. First, the precise control of electrical stimulation is challenging due to the complexity of olfactory processing, which involves multiple brain regions interconnected in complex non-linear ways. Unlike other senses, olfactory information bypasses the thalamus and directly connects to areas responsible for emotions and memory, such as the amygdala and hippocampus (Wilson and Mainen, 2006). This unique pathway complicates the task of delivering targeted stimulation without unintended effects on mood and cognition. Additionally, individual variability in olfactory perception (Hudson and Distel, 2002) means that the same stimulation could produce different results across subjects, further complicating the development of standardized protocols for inducing smell.

The other limitation lies in the technical aspects of delivering non-invasive electrical stimulation. Recent advances have made progress in addressing this issue. Cakmak et al. (2020) optimized electrode placements for non-invasive electrical stimulation of both the olfactory bulb and olfactory mucosa, demonstrating a more precise approach to targeting these structures without invasive procedures. This development is significant because it could help mitigate some of the risks associated with invasive techniques, such as transethmoidal stimulation of the olfactory bulb (Holbrook et al., 2019). Nevertheless, the persistence of olfactory stimulation and the complexity of odor mixtures make it difficult to control and modulate induced smells in real-time, limiting the applicability of such techniques in dynamic environments like virtual reality or everyday life (Yanagida, 2012).

In conclusion, we are at the beginning of the development of olfactory-based BCIs and O-NFB systems, and the potential for such devices appears to be considerable.

## Data Availability

The original contributions presented in the study are included in the article/supplementary material, further inquiries can be directed to the corresponding author.
